# Associations of leptin and adiponectin with incident type 2 diabetes and interactions among African Americans: the Jackson heart study

**DOI:** 10.1186/s12902-020-0511-z

**Published:** 2020-03-04

**Authors:** Aurelian Bidulescu, Paul C. Dinh, Shabir Sarwary, Emily Forsyth, Maya C. Luetke, David B. King, Jiankang Liu, Sharon K. Davis, Adolfo Correa

**Affiliations:** 1 Department of Epidemiology and Biostatistics, Indiana University School of Public Health, 1025 E. 7th Street, Bloomington, IN 47405 USA; 2 Henry M Jackson Foundation for the advancement of Military Medicine, Bethesda, MD USA; 3 Brigham and Women’s Hospital, Boston, MA USA; 4 National Human Genome Research Institute, Genomics of Metabolic, Cardiovascular and Inflammatory Disease Branch, Social Epidemiology Research Unit, Bethesda, MD USA; 5 Jackson Heart Study at University of Mississippi Medical Center, Jackson, MS USA

**Keywords:** Leptin, Adiponectin, Type 2 diabetes, African Americans, Insulin resistance, Mediation, Observational cohort, Obesity, Gender

## Abstract

**Background:**

Growing evidence suggests that leptin is critical for glycemic control. Impaired leptin signaling may also contribute to low adiponectin expression in obese individuals. We assessed the association of leptin and adiponectin with incident type 2 diabetes (T2D), their interactions with sex and obesity status, and mediation by insulin resistance.

**Methods:**

We included study participants from the Jackson Heart Study, a prospective cohort of adult African Americans in Jackson, Mississippi, that were free of T2D at the baseline Exam 1. Incident T2D was defined as new cases at Exam 2 or Exam 3. We created separate Cox regression models (hazard ratios per log-transformed ng/mL of leptin and adiponectin) with and without insulin resistance, HOMA-IR. Mediation by insulin resistance was analyzed. Several interactions were assessed, including by sex, HbA1c, and obesity.

**Results:**

Among our 3363 participants (mean age 53 years, 63% women), 584 developed incident T2D. Leptin was directly associated with incident T2D when modeled without HOMA-IR (HR = 1.29, 95% CI = 1.05–1.58). This direct association between leptin and T2D was significant among men (HR = 1.33, 95% CI = 1.05–1.69), but nonsignificant among women (HR = 1.24, 95% CI = 0.94–1.64); statistical interaction with sex was nonsignificant (*p* = 0.65). The associations in all participants and in men were nullified by HOMA-IR (HR = 0.99, 95% CI = 0.80–1.22; HR = 1.00, 95% CI = 0.78–1.28, respectively), indicating mediation through insulin resistance (proportion mediated: 1.04), and were not observed in abdominally obese participants. Adiponectin was inversely associated with T2D even after adjustment for HOMA-IR in women (HR = 0.68, 95% CI = 0.55–0.84), but not in men (HR = 0.80, 95% CI = 0.62–1.04). The inverse association was present only among abdominally obese participants, and persisted after adjustment for HOMA-IR.

**Conclusions:**

Among African Americans in the Jackson Heart Study the association of leptin with incident type 2 diabetes was mediated by insulin resistance. This association was present only among abdominally non-obese participants. Differences by sex appeared: men showed a significant association mediated by insulin resistance. Among abdominally obese participants, adiponectin was inversely associated with incident T2D even after adjustment for HOMA-IR. Our results should inform future clinical trials that aim to reduce the burden of type 2 diabetes through the modification of serum levels of leptin and adiponectin.

## Background

The burden of type 2 diabetes remains high in the United States and globally despite advances in medicine and technology such as anti-diabetic drugs and glucose monitoring systems [1]. Since 1980, the number of adults with diagnosed type 2 diabetes in the U.S. has more than quadrupled from 5.5 million to almost 30.3 million in 2017 [2, 3]. Among African American adults, the prevalence of type 2 diabetes has increased from 9.7% in 1997 to 12.6% in 2012 [4].

While it is well established that the adiposity hormone leptin plays a key role in body weight regulation and energy homeostasis, [5, 6] growing evidence suggests that leptin may also be critical for glycemic control independent of its effects on calorie consumption and energy expenditure [7–10]. However, the evidence in human studies suggesting leptin’s role is independent from its actions on body weight was observed in lipodystrophic individuals where leptin replacement therapy reversed insulin resistance [8–11]. The lack of adipose tissue in these individuals results in lipotoxicity-induced insulin resistance and low levels of leptin (fasting leptin concentration of less than 4 ng/ml) [8–11]. As such these results may not be generalizable to individuals with normal levels of leptin. In population studies, high leptin levels were associated with the development of diabetes, with differences by sex [5, 6]. In mouse models of type 2 diabetes, leptin improved insulin resistance and hyperglycemia [12]. Moreover, research has also shown that leptin and adiponectin, another adiposity hormone, may be linked. Impaired leptin signaling may contribute to low adiponectin expression in obese individuals. As such, increasing leptin signaling may be a target for interventions aiming to increase adiponectin expression, enhance insulin sensitivity, and improve the cardiometabolic profile in obesity [13]. Because individuals with a greater amount of abdominal fat have relatively low leptin concentrations, which in turn relates to a metabolic profile compatible with an increased cardiovascular risk, measures of body fat distribution are essential in the assessment of the relationship between leptin and type 2 diabetes.

As African Americans have a higher prevalence of both obesity and diabetes, we aimed to assess the association of leptin with type 2 diabetes in the Jackson Heart Study, the largest ongoing cohort of African Americans. We hypothesized that in this sample, there would be a significant relationship between higher leptin levels and an individual’s risk of type 2 diabetes with differences by sex. We also aimed to assess if the relationship between leptin levels and type 2 diabetes risk was modified by adiponectin levels, sex, obesity status, and fat distribution.

## Methods

### Study subjects

The Jackson Heart Study is a single-site, prospective cohort study of risk factors and causes of heart disease in adult African Americans in three contiguous counties (Hinds, Madison, and Rankin) surrounding Jackson, Mississippi. Between 2000 and 2004, 5306 African Americans aged 21–94 years were recruited, examined, and enrolled in the Jackson Heart Study by certified technicians according to standardized protocols at baseline, using a self-administered questionnaire, an in-home interview, and a clinic visit (Exam 1) [14, 15]. Two follow-up clinical examinations at 4-year intervals were conducted between 2005 and 2008 (Exam 2) and between 2009 and 2012 (Exam 3). The present study included data from 3363 participants. Participants were excluded if they had type 2 diabetes at the baseline examination (*n* = 1152), were missing types 2 diabetes status at the baseline Exam 1 (*n* = 66), or were missing type 2 diabetes status at both follow-up Exam 2 and Exam 3 (*n* = 725). Regression analyses used complete-case data from 2656 participants due to missing values for adiponectin, leptin, BMI, waist circumference, systolic blood pressure, antihypertension medication use, triglycerides, HDL-cholesterol, anti-hyperlipidemic medication use, smoking, alcohol intake, physical activity, education level, and HOMA-IR. Blood pressure, anthropometry, medical history, cardiovascular risk factors, blood samples, and urine samples were measured or collected at the baseline examination. Additionally, each participant filled out a written consent form to participate in the Jackson Heart Study and the Institutional Review Boards of the National Institutes of Health and the Jackson Heart Study institutions (the University of Mississippi Medical Center, Tougaloo College, and the University of Mississippi Medical Center) approved the study protocol.

### Outcome variable

Incident type 2 diabetes was defined as new cases of type 2 diabetes at Exam 2 or Exam 3 among participants who did not have type 2 diabetes at baseline exam, Exam 1 (584 incident cases). Type 2 diabetes was defined as HbA1c ≥ 6.5% (48 mmol/mol), fasting blood glucose ≥126 mg/dL, self-reported use of insulin or oral hypoglycemic medications, or a physician diagnosis of the condition.

### Exposure variables

Plasma leptin concentrations were derived at baseline using venous blood samples from each participant after more than 8 h of fasting. Samples were stored at − 80 °C in the Jackson Heart Study central repository. They were analyzed with Human Leptin PIA kit [16]. The coefficient of variation was 10%, indicating the sample had not undergone significant biological degradation and would yield valid measures of leptin in the blood [16]. Leptin levels were log-transformed to normalize the distribution before analyses. Sex-specific quartiles were also created. For men, the leptin quartiles were defined as: (Q1) < 4.5 ng/mL, (Q2) ≥4.5 to < 7.8 ng/mL, (Q3) ≥7.8 to < 13.3 ng/mL, and (Q4) ≥13.3 ng/mL. For women, the leptin quartiles were defined as: (Q1) < 21.85 ng/mL, (Q2) ≥21.85 to < 32.9 ng/mL, (Q3) ≥32.9 to < 46.3 ng/mL, and (Q4) ≥46.3 ng/mL.

Adiponectin concentrations were also derived via venous blood samples drawn from each participant after more than 8 h of fasting. Measurements were made in 2009–2010 as total circulating plasma adiponectin using an ELISA system [15, 17]. The inter-assay coefficient of variation was 8.8%, again indicating no biological degradation of stored specimens and a high validity for measurement [18]. Adiponectin levels were also log-transformed to normalize the distribution before analyses. Additionally, sex-specific quartiles were also created. For men, the adiponectin quartiles were defined as: (Q1) < 2066.200 ng/mL, (Q2) ≥2066.200 to < 3118.041 ng/mL, (Q3) ≥3118.041 to < 4892.741 ng/mL, and (Q4) ≥4892.741 ng/mL. For women, the adiponectin quartiles were defined as: (Q1) < 3327.154 ng/mL, (Q2) ≥3327.154 to < 5080.525 ng/mL, (Q3) ≥5080.525 to < 7606.885 ng/mL, and (Q4) ≥7606.885 ng/mL.

### Covariates

All covariate variables were collected at baseline and were chosen because they are known risk factors for type 2 diabetes and/or associated with adiponectin/leptin [19]. These covariates included three different types of variables: demographic, biological, and behavioral.

Demographic covariates included sex, age, and education level. Sex was a dichotomous variable of male versus female. Age was derived at baseline from date of birth and maintained as a continuous variable. Education level was defined as a four-category variable: (1) less than high school, (2) high school graduate, (3) some college, and (4) bachelor’s degree or more.

Biological risk factor measures included anti-hypertriglyceridemic medication use, anti-hyperlipidemic medication use, anti-hypertensive medication use, systolic blood pressure, fasting insulin, low-density lipoprotein cholesterol (LDL), high-density lipoprotein cholesterol (HDL), total cholesterol (TC), triglyceride, lipoprotein(a), fasting glucose, fasting insulin, HbA1c, and homeostasis model assessment–insulin resistance (HOMA-IR). Fasting insulin, fasting glucose, HbA1c, LDL, HDL, lipoprotein(a), and TC were assessed using standard laboratory techniques. Insulin resistance status was estimated with the HOMA model assessment [20]. The use of antihypertriglyceridemic, antihyperlipidemic, and antihypertensive medications were each defined as “yes” for use at baseline or “no” for no use at baseline.

Behavioral risk factor variables included smoking status, physical activity, alcohol status, and body mass index (BMI). Smoking status was defined as former, current, and never smokers. Physical activity was assessed with a four-domain physical activity survey instrument: (1) active living, (2) work, (3) home and garden, and (4) sport and exercise indexes. These scores ranged from 1 to 5, with higher scores indicating a higher level of physical activity. The sum of these four domain scores was used to calculate a total score with a maximum value of 20 [19]. Alcohol consumption was categorized as abstaining, < 1 drink/week, 1–7 drinks/week, 8–14 drinks/week, > 14 drinks/week in the past 12 months. Body mass index (BMI) measurements were based on standing height and weight measured in lightweight clothing without shoes or constricting garments and calculated as weight in kilograms divided by height in meters squared (kg/m2). For analyses, BMI was categorized by current standard cut points: (1) < 25 kg/m2 (normal and underweight), (2) ≥25 - < 30 kg/m2 (overweight), and (3) ≥30 kg/m2 (obese). Waist circumference was measured to the nearest centimeter at the navel. For analyses, waist circumference was also dichotomized according to standard sex-specific abdominal obesity cut points. Men with waist circumference ≥ 102 cm and women with waist circumference ≥ 88 cm were considered abdominally obese while men with waist circumference < 102 cm and women with waist circumference < 88 cm were considered abdominally non-obese.

Based upon past literature and to avoid issues of collinearity, we included the following variables as confounders in our models: sex, age, BMI, waist circumference, systolic blood pressure, antihypertensive medication, triglycerides, HDL-cholesterol, antihyperlipidemic medication, smoking, alcohol intake, physical activity, education level, and HOMA-IR.

### Statistical analysis

Descriptive analyses for all the above variables were produced. T-tests or one-way analyses of variance (ANOVA) were used to assess the bivariate relationships between continuous adiponectin and leptin variables and categorical covariates. Chi-square tests were used to assess the bivariate analysis between quartiles of adiponectin and leptin and categorical covariates. Pearson correlations were used to assess the association between continuous adiponectin and leptin versus continuous covariates. The association of quartiles of adiponectin and leptin with continuous covariates was assessed with one-way ANOVA. Finally, bivariate analyses of incident type 2 diabetes with continuous and categorical covariates were assessed with t-tests or Chi-square tests, respectively. Log transformations were applied to all non-normally distributed continuous variables prior to further analysis.

Separate Cox proportional hazard regression models were utilized to model the risk of incident type 2 diabetes. Model 1 included both adiponectin and leptin and adjusted additionally for sex, age, BMI, waist circumference, systolic blood pressure, antihypertension medication, triglycerides, HDL-cholesterol, antihyperlipidemic medication, smoking, alcohol intake, physical activity and education level. Model 2 included HOMA-IR in addition to all variables from Model 1. Date of incidence of type 2 diabetes was estimated as the midpoint between the first examination date with type 2 diabetes and the prior examination date. Time of follow-up was from the first exam date until the date of incident type 2 diabetes or lost to follow-up.

The interaction of leptin and adiponectin were assessed with two-way interaction terms. Separate models were created for the interaction between log-transformed leptin and log-transformed adiponectin, and between quartiles of leptin and quartiles of adiponectin. The effect measure modification of sex with log-transformed adiponectin and log-transformed leptin was assessed using two-way interaction terms. This was repeated for high HbA1c (greater than 5.7%) with log-transformed adiponectin and log-transformed leptin. Similar two-way interaction models for sex and high HbA1c were created for quartiles of adiponectin and quartiles of leptin. Level specific *P*-values for quartiles of leptin and quartiles of adiponectin were assessed via Wald tests for multiple comparison contrasts. Additional models were stratified by BMI groups. Similar interaction tests were repeated for BMI groups. Models were also stratified by waist circumference risk groups and interaction tests repeated.

Mediation analysis was conducted for Cox models as demonstrated by Valeri et al. [21, 22] Mediation analysis attempts to resolve how much of an observed change in an outcome is attributable to a change in an exposure and how much is due to the change in a mediator. Natural direct, natural indirect, and total effects were estimated without an exposure-mediator interaction. The natural direct effect expresses how much the outcome would change if the exposure were changed from level A* to level A. The natural indirect effect expresses how much the outcome would change on average if the exposure were controlled at level A. The total effect can be defined as how much the outcome would change overall for a change in the exposure from level A* to level A [21, 22]. Natural direct, natural indirect, and total effects were estimated for a 1 unit increase from A* to A. These values were used to estimate the proportion mediated [21, 22].

A two-tailed level of significance was established as *P* < .05. Analyses were conducted using SAS version 9.4 (SAS Institute Inc., Cary, North Carolina).

## Results

The mean follow-up was 2581 days (7.07 years), and the median follow-up was 2742 days (7.51 years). For those with incident type 2 diabetes, the mean follow-up was 1408 days (3.85 years), and the median follow-up was 1201 days (3.29 years). For those without incident type 2 diabetes, the mean follow-up was 2828 days (7.74 years), and the median follow-up was 2877 days (7.88 years). The bivariate analyses revealed significant associations between incident type 2 diabetes and education level, anti-hyperlipidemic medication use, age, physical activity, systolic blood pressure, BMI, waist circumference, HDL cholesterol, triglyceride, HOMA-IR, fasting insulin, fasting glucose, HbA1c, plasma leptin, and plasma adiponectin (Table 1). Participants with incident type 2 diabetes were older, less educated, prescribed more anti-hyperlipidemic medication, and less physically active. These participants also had higher systolic blood pressure, BMI, waist circumference, triglycerides, HOMA-IR, fasting insulin, fasting glucose, HbA1c, and plasma leptin, but lower HDL cholesterol and plasma adiponectin (Table 1). Leptin levels were higher in women than men in those with diabetes (women: mean = 43.4 ng/ml, median = 38.0 ng/ml, IQR = 27.5–52.0 ng/ml; men: mean = 13.3 ng/ml, median = 10.7 ng/ml, IQR = 6.1–16.7 ng/ml) and in those without diabetes (women: mean = 35.4 ng/ml, median = 31.7 ng/ml, IQR = 20.8–45.2 ng/ml; men: mean = 9.8 ng/ml, median = 7.1 ng/ml, IQR = 4.3–12.1 ng/ml).

**Table 1:** Participant characteristics by incident *type 2 diabetes* status

Characteristic	n	Incident type 2 diabetes		*P*-value*
		Yes (*n* = 584)	No (*n* = 2779)	
Sex, n (%)				***0.82***
Male	1230	216 (37.0%)	1014 (36.5%)	
Female	2133	368 (63.0%)	1765 (63.5%)	
Total	3363	584	2779	
Education Level, n (%)				***<.01***
Less than high school	459	102 (17.5%)	357 (12.9%)	
High school graduate	645	121 (20.8%)	524 (18.9%)	
Some college	1002	174 (29.9%)	828 (29.8%)	
Bachelor’s degree or higher	1250	185 (31.8%)	1065 (38.4%)	
Total	3356	582	2774	
Smoking Status, n (%)				***0.07***
Never	2368	388 (67.6%)	1980 (71.7%)	
Former	569	116 (20.2%)	453 (16.4%)	
Current	397	70 (12.2%)	327 (11.8%)	
Total	3334	574	2760	
Alcohol Consumption, n (%)				***0.20***
Abstaining	1678	316 (55.1%)	1362 (50.4%)	
< 1 drink/week	858	147 (25.7%)	711 (26.3%)	
1–7 drinks/week	519	78 (14.0%)	441 (16.8%)	
8–14 drinks/week	126	17 (3.0%)	109 (4.0%)	
> 14 drinks/week	96	15 (2.6%)	81 (3.0%)	
Total	3277	573	2704	
Anti-hypertensive Medication, n (%)				***<.0001***
Yes	1404	326 (59.9%)	1078 (41.2%)	
No	1756	218 (40.1%)	1538 (58.8%)	
Total	3160	544	2616	
Anti-hypertriglyceridemic Medication, n (%)				***0.30***
Yes	6	2 (0.4%)	4 (0.2%)	
No	3081	531 (99.6%)	2550 (99.8%)	
Total	3087	533	2554	
Anti-hyperlipidemic Medication, n (%)				***< 0.01***
Yes	275	66 (12.4%)	209 (8.2%)	
No	2812	467 (87.6%)	2345 (91.8%)	
Total	3087	533	2554	
Age (years), mean ± se	3363	54.7 ± 0.5	52.5 ± 0.2	***<.0001***†
Physical activity score, mean ± se	3183	8.4 ± 0.1	8.8 ± 0.05	***<.001***
Systolic blood pressure (mmHg), mean ± se	3354	128.0 ± 0.7	124.4 ± 0.3	***<.0001***
BMI (kg/m^2^), mean ± se	3360	33.6 ± 0.3	30.7 ± 0.1	***<.0001***
Waist (cm), mean ± se	3360	104.9 ± 0.6	97.3 ± 0.3	***<.0001***†
Male	1228	104.9 ± 0.9	98.4 ± 0.5	
Female	2132	104.9 ± 0.8	96.6 ± 0.4	
LDL-Cholesterol (mg/dL), mean ± se	3326	128.8 ± 1.6	127.0 ± 0.7	***0.29***
HDL-Cholesterol (mg/dL), mean ± se	3339	49.2 ± 0.5	52.8 ± 0.3	***<.0001***†
Male	1226	44.0 ± 0.7	47.0 ± 0.4	
Female	2113	52.3 ± 0.7	56.1 ± 0.3	
Total Cholesterol (mg/dL), mean ± se	3339	200.9 ± 1.7	198.7 ± 0.7	***0.24***†
Triglyceride (mg/dL), mean ± se	3339	114.3 ± 2.5	95.2 ± 1.0	***<.0001***†
Lipoprotein(a), mean ± se	3256	58.0 ± 2.0	58.4 ± 0.8	***0.83***
HOMA-IR, mean ± se	3210	4.9 ± 0.1	3.3 ± 0.04	***<.0001***†
Fasting insulin (ug/mL), mean ± se	3343	20.8 ± 0.5	14.7 ± 0.2	***<.0001***†
Fasting Glucose (mg/dL), mean ± se	3238	97.1 ± 0.4	88.9 ± 0.2	***<.0001***†
HbA1c, mean ± se	3286	5.9 ± 0.0	5.4 ± 0.01	***<.0001***†
Circulating Plasma leptin (ng/mL), mean ± se	3319	32.1 ± 1.1	26.0 ± 0.4	***<.0001***†
Male	1219	13.3 ± 0.7	9.8 ± 0.3	
Female	2100	43.4 ± 1.5	35.4 ± 0.5	
Circulating Plasma leptin, n (%)‡				***<.0001***
Quartile 1	804	75 (13.0%)	729 (26.6%)	
Quartile 2	835	136 (23.5%)	699 (25.5%)	
Quartile 3	839	149 (25.7%)	690 (25.2%)	
Quartile 4	841	219 (37.8%)	622 (22.7%)	
Total	3319	579	2740	
Circulating Plasma Adiponectin (ng/mL), mean ± se	3299	4091.9 ± 115.1	5566.3 ± 77.2	***<.0001***†
Male	1215	3202.9 ± 148.6	4061.0 ± 94.6	
Female	2084	4624.8 ± 154.5	6438.7 ± 103.3	
Circulating Plasma Adiponectin, n (%)§				***<.0001***
Quartile 1	849	221 (38.7%)	628 (23.0%)	
Quartile 2	840	161 (28.2%)	679 (24.9%)	
Quartile 3	831	118 (20.7%)	713 (26.1%)	
Quartile 4	779	71 (12.4%)	708 (26.0%)	
Total	3299	571	2728	

*T-tests were used for continuous variables and Chi-square tests for categorical variables

†Satterthwaite test for unequal variances

‡For men the sex-specific leptin quartiles were defined as < 4.5 ng/mL (Q1), ≥4.5 to < 7.8 ng/mL (Q2), ≥7.8 to < 13.3 ng/mL (Q3), ≥13.3 ng/mL (Q4). For women, the leptin quartiles were defined as < 21.85 ng/mL (Q1), ≥21.85 to < 32.9 ng/mL (Q2), ≥32.9 to < 46.3 ng/mL (Q3), ≥46.3 ng/mL (Q4)

§For men the sex-specific adiponectin quartiles were defined as < 2066.200 ng/mL (Q1), ≥2066.200 to < 3118.041 ng/mL (Q2), ≥3118.041 to < 4892.741 ng/mL (Q3), ≥4892.741 ng/mL (Q4). For women the adiponectin quartiles were defined as < 3327.154 ng/mL (Q1), ≥3327.154 to < 5080.525 ng/mL (Q2), ≥5080.525 to < 7606.885 ng/mL (Q3), ≥7606.885 ng/mL (Q4)

### Incident type 2 diabetes risk

Cox proportional hazard regression models of incident type 2 diabetes showed a significant association between leptin, adiponectin, and incident type 2 diabetes in Model 1 (*P* = 0.015 and *P* < 0.0001, respectively) (Table 2). Higher leptin levels were associated with increased risk of incident type 2 diabetes in Model 1(HR = 1.29; 95% CI = 1.05–1.58, P = 0.015), while higher adiponectin levels were associated with decreased risk (HR = 0.57; 95% CI = 0.49–0.67; P < 0.0001). However, the fully-adjusted HOMA-IR model (Model 2) estimated no significant association between higher leptin levels and incident type 2 diabetes (HR = 0.99; 95% CI = 0.80, 1.22; *P* = 0.92) but did show significance with adiponectin (HR = 0.73; 95% CI = 0.61, 0.86; *P* = 0.0003) (Table 2). To investigate a possible mediator role of HOMA-IR with leptin and type 2 diabetes, post hoc mediation analysis was performed. The estimated proportion mediated was 1.04. The natural direct effect hazard ratio was 0.99 (95% CI = 0.80–1.22), the natural indirect effect hazard ratio was 1.29 (95% CI = 1.21–1.37) and the total effect hazard ratio was 1.27 (95% CI = 1.03–1.57). Thus, insulin resistance, measured by HOMA-IR, mediated the association between leptin levels and incident type 2 diabetes. In Model 1, the hazard ratio of type 2 diabetes increased as quartiles of leptin increased, when compared to the reference first quartile (data not shown). Adiponectin was inversely associated with type 2 diabetes in all models (Table 2). The hazard ratio of type 2 diabetes decreased as the quartiles of adiponectin increased when compared to the reference first quartile (data not shown). No effect modification was evident between quartiles of leptin and quartiles of adiponectin in Models 1 and 2 (*P* = 0.67, *P* = 0.59, respectively, data not shown) and, similarly, between log leptin and log adiponectin in Models 1 and 2 (*P* = 0.10, 0.34, respectively, data not shown).

**Table 2:** Prospective associations (hazard ratios) between leptin and adiponectin with incident *type 2 diabetes* in the total sample population and by sex

	*Total*				*Sex*							
	*Sample Population (n = 2656)* *Type 2 diabetes cases (n = 455)*				*Male (n = 948)* *Type 2 diabetes cases (n = 169)*				*Female (n = 1708)* *Type 2 diabetes cases (n = 286)*			
***Continuous Adipokines***	Model 1*		***Model 2***†		Model 1*		***Model 2***†		Model 1*		***Model 2***†	
	**HR**	95% CI	**HR**	95% CI	**HR**	95% CI	**HR**	95% CI	**HR**	95% CI	**HR**	95% CI
Log Leptin (ng/mL)	**1.29**	(1.05, 1.58)	**0.99**	(0.80, 1.22)	**1.33**	(1.05, 1.69)	**1.00**	(0.78, 1.28)	**1.24**	(0.94, 1.64)	**0.99**	(0.74, 1.31)
Log Adiponectin (ng/mL)	**0.57**	(0.49, 0.67)	**0.73**	(0.61, 0.86)	**0.68**	(0.52, 0.88)	**0.80**	(0.62, 1.04)	**0.52**	(0.42, 0.63)	**0.68**	(0.55, 0.84)

*Model 1: Adjusted for sex, age, BMI, waist circumference, systolic blood pressure, anti-hypertensive medication, triglycerides, HDL-cholesterol, antihyperlipidemic medication, smoking, alcohol consumption, physical activity, and education level

†Model 2: Adjusted for model 1 variables and HOMA-IR

### Interaction effects

Table 2 also presents the hazard ratios of the sex-leptin interaction and sex-adiponectin interaction for type 2 diabetes. Though the interaction term was not significant (*P* = 0.65) in Model 1, the association between leptin and type 2 diabetes was significant in men (HR = 1.33; 95% CI = 1.05–1.69) but not in women (HR = 1.24; 95% CI = 0.94–1.64). In Model 2, leptin was not significantly associated with type 2 diabetes in both men and women. In men, the estimated proportion mediated was 0.84. The natural direct effect hazard ratio was 1.12 (95% CI = 0.80–1.57), the natural indirect effect hazard ratio was 1.38 (95% CI = 1.22–1.57), and the total effect hazard ratio was 1.55 (95% CI = 1.12–2.15). This result again shows the mediation of leptin and type 2 diabetes through insulin resistance. In women, the estimated proportion mediated was 1.67. The natural direct effect hazard ratio was 0.94 (95% CI = 0.69–1.28), the natural indirect effect hazard ratio was 1.24 (95% CI = 1.15–1.33) and the total effect hazard ratio was 1.16 (95% CI = 0.86–1.57). The non-significant association between leptin and type 2 diabetes in women was entirely mediated by insulin resistance. Adiponectin was inversely associated with type 2 diabetes even after adjustment for HOMA-IR among women (HR = 0.68; 95% CI = 0.55–0.84), but not among men (HR = 0.80; 95% CI = 0.62–104).

### Stratification by obesity

Stratification by BMI group showed no significant association with leptin and incident type 2 diabetes in Models 1 or 2 for any of the 3 BMI groups (Table 3). Adiponectin was associated with type 2 diabetes for those with BMI ≥ 30 kg/m2 in Models 1 (HR = 0.54; 95% CI = 0.44, 0.66) and 2 (HR = 0.69; 95% CI = 0.56, 0.86; Table 3). No interaction tests were significant (data not shown. Effect modification was not present between either log-transformed or quartiles of either leptin or adiponectin and HbA1c dichotomized at 5.7% (data not shown). Stratification by abdominal obesity groups showed similar results. Leptin was significantly associated with incident type 2 diabetes (HR = 1.55; 95% CI = 1.02, 2.36) among the abdominally non-obese, which became non-significant after adjustment for HOMA-IR (Table 4). Adiponectin was inversely associated with type 2 diabetes only among abdominally obese participants, which remained significant after adjustment for HOMA-IR (HR = 0.66; 95% CI = 0.54, 0.80; Table 4). Again, interaction tests were not significant (data not shown).

**Table 3:** Prospective associations (hazard ratios) between leptin and adiponectin with incident *type 2 diabetes* stratified by BMI groups

*BMI < 25 kg/m*^*2*^ *(n = 416)*						
*Type 2 diabetes cases (n = 28)*						
***Continuous Adipokines***	Model 1*			***Model 2***†		
	**HR**	95% CI	*P*	**HR**	95% CI	*P*
Log Plasma leptin (ng/mL)	**1.42**	(0.65, 3.10)	*0.38*	**1.14**	(0.50, 2.60)	*0.75*
Log Plasma adiponectin (ng/mL)	**1.01**	(0.49, 2.10)	*0.98*	**1.10**	(0.52, 2.33)	*0.80*
*BMI ≥ 25 - < 30 kg/m*^*2*^ *(n = 929)*						
*Type 2 diabetes cases (n = 119)*						
***Continuous Adipokines***	Model 1*			***Model 2***†		
	**HR**	95% CI	*P*	**HR**	95% CI	*P*
Log Plasma leptin (ng/mL)	**1.33**	(0.89, 2.01)	*0.17*	**1.00**	(0.65, 1.53)	*0.99*
Log Plasma adiponectin (ng/mL)	**0.63**	(0.46, 0.87)	*<.01*	**0.77**	(0.55, 1.08)	*0.13*
*BMI ≥ 30 kg/m*^*2*^ *(n = 1311)*						
*Type 2 diabetes cases (n = 308)*						
***Continuous Adipokines***	Model 1*			***Model 2***†		
	**HR**	95% CI	*p*	**HR**	95% CI	*P*
Log Plasma leptin (ng/mL)	**1.09**	(0.83, 1.42)	*0.55*	**0.88**	(0.66, 1.16)	*0.37*
Log Plasma adiponectin (ng/mL)	**0.54**	(0.44, 0.66)	*<.0001*	**0.69**	(0.56, 0.86)	*<.001*

*Model 1: Adjusted for sex, age, waist circumference, systolic blood pressure, antihypertension medication, triglycerides, HDL-cholesterol, antihyperlipidemic medication, smoking, alcohol consumption, physical activity, and education level

†Model 2: Adjusted for model 1 variables and HOMA-IR

**Table 4:** Prospective associations (hazard ratios) between leptin and adiponectin with incident *type 2 diabetes* stratified by abdominal obesity (as measured by waist circumference*)

*Abdominally non-obese (n = 1057)*						
*Type 2 diabetes cases (n = 91)*						
***Continuous Adipokines***	***Model 1***†			***Model 2***‡		
	**HR**	95% CI	*P*	**HR**	95% CI	*P*
Log Plasma leptin (ng/mL)	**1.55**	(1.02, 2.36)	*0.04*	**0.92**	(0.58, 1.45)	*0.73*
Log Plasma adiponectin (ng/mL)	**0.86**	(0.60, 1.23)	*0.40*	**1.18**	(0.80, 1.73)	*0.41*
*Abdominally obese (n = 1599)*						
*Type 2 diabetes cases (n = 364)*						
***Continuous Adipokines***	***Model 1***†			***Model 2***‡		
	**HR**	95% CI	*p*	**HR**	95% CI	*P*
Log Plasma leptin (ng/mL)	**1.04**	(0.81, 1.34)	*0.75*	**0.82**	(0.63, 1.07)	*0.14*
Log Plasma adiponectin (ng/mL)	**0.53**	(0.44, 0.63)	*<.0001*	**0.66**	(0.54, 0.80)	*<.0001*

*Waist circumference was dichotomized with sex-specific cut points as abdominally obese (men with waist circumference ≥ 102 cm and women with waist circumference ≥ 88 cm) and abdominally non-obese (men with waist circumference < 102 cm and women with waist circumference < 88 cm)

†Model 1: Adjusted for sex, age, BMI, systolic blood pressure, antihypertension medication, triglycerides, HDL-cholesterol, antihyperlipidemic medication, smoking, alcohol consumption, physical activity, and education level

‡Model 2: Adjusted for model 1 variables and HOMA-IR

## Discussion

Our investigation among African Americans in the Jackson Heart Study revealed an association between both leptin and adiponectin and incident type 2 diabetes mellitus. We found an increased risk of incident type 2 diabetes with increasing leptin levels. In contrast, higher adiponectin levels decreased the risk of incident type 2 diabetes. Further, insulin resistance mediated the association between leptin and incident type 2 diabetes. Differences by sex were also present, with male participants showing an association between leptin and incident type 2 diabetes mediated by insulin resistance. There was no interaction between adiponectin and leptin in their association with incident type 2 diabetes. Similarly, no interaction with HbA1c or overall obesity was present. However, the association between leptin and type 2 diabetes was completely attenuated among abdominally obese participants. Among abdominally obese participants, adiponectin was inversely associated with incident T2D even after adjustment for HOMA-IR.

There is growing evidence from animal models that leptin and adiponectin play a critical role in type 2 diabetes prevention and control by promoting beta-cell function and survival, improving insulin sensitivity, and regulating glucose metabolism [23–26]. Insulin resistance in lipoatrophic mice was completely reversed by combining doses of adiponectin and leptin but was only partially reversed by either adiponectin or leptin alone [27]. Leptin and adiponectin regulate blood glucose by several different mechanisms. Leptin’s effects on appetite and fat storage are mediated by leptin receptors on neurons in the central nervous system. These signals suppress food intake and permit energy expenditure. Additionally, leptin’s effects on obesity and insulin resistance are mediated by pro-opiomelanocortin (POMC) [28, 29]. Studies using mouse models have shown that restoration of leptin receptor expression in hypothalamic arcuate nucleus (ARH) neurons normalizes hyperglycemia in obese and diabetic mice. Thus, ARH POMC neurons are beneficial for mediating the anti-diabetic actions of leptin in diabetic mice [29]. As for adiponectin, this highest-level fat hormone acts as an anti-inflammatory hormone with insulin-sensitizing properties [30]. Moreover, adiponectin affects the pancreas by promoting beta cell function and survival and increases glucose-mediated insulin secretion in high-fat-diet-fed mice [26, 30]. Adiponectin decreases triglyceride content in muscle and liver, which leads to increased expression of molecules involved in both fatty-acid combustion and energy dissipation [27]. Therefore, adiponectin increases fatty acid oxidation while reducing the synthesis of glucose in the liver via two receptors [31]. In obese individuals, impaired leptin signaling may contribute to low adiponectin expression. Thus, leptin signaling may be a target to increase adiponectin expression, improve insulin sensitivity, and improve the cardio-metabolic profile. Though our results did not show an interaction between adiponectin and leptin towards type 2 diabetes, it does not preclude the possibility that adiponectin and leptin are mediators on the same pathway. Our findings of an inverse association of adiponectin with incident type 2 diabetes and a positive association of leptin with incident type 2 diabetes mediated through insulin resistance corroborate findings from cross-sectional studies while adding a prospective/longitudinal element.

Observational studies have indicated that African Americans have lower levels of adiponectin, lower socioeconomic status, higher levels of type 2 diabetes and poorer lifestyle profiles compared to other racial groups in the U.S. A study conducted in the Atherosclerosis Risk in Communities (ARIC) cohort found African Americans had 22% lower mean values for adiponectin when compared to whites [32]. In that same study, women had 52% higher mean values for adiponectin [32]. Previous findings from the Jackson Heart Study have shown an inverse association between adiponectin and type 2 diabetes among women, and no significant association among men in both crude and adjusted multivariate models [19].

There is a strong correlation between circulating leptin levels, fat mass, and resting metabolic rate in healthy men and women [33]. Nevertheless, some previous studies have shown no significant correlations between leptin and body fat parameters among men, while among women, serum leptin concentrations were highly correlated with body fat distribution and mean fat cell size [34]. Their leptin concentrations were also almost four times higher than in men [34]. In this study, the direct association between leptin and incident type 2 diabetes observed among abdominally non-obese participants supports previous findings that about 40% of the variation in leptin in obese subjects could be explained by peripheral fat distribution [35]. Studies have shown that leptin plasma concentrations are dependent on body fat distribution in obese patients [36] and that there is a negative correlation between plasma leptin and waist-to-hip ratio, independent of BMI [35]. Additionally, leptin, but not adiponectin, was correlated with both waist and hip circumference, [37] which refutes findings from a study published in 1996 that showed plasma leptin concentrations were highly correlated with body fat percentage [38]. In this same study, leptin was not independently related to abdominal fat distribution and the authors concluded that circulating leptin climbs continuously with increasing adiposity [38]. A more recent study showed a strong positive association between subcutaneous adipose tissue and leptin levels, but not visceral adipose tissue nor liver fat content and leptin, independent of the HOMA-IR status [39]. Our finding of increased risk of type 2 diabetes with increased levels of leptin observed among abdominally non-obese participants adds weight to the “leptin resistance’ phenomenon in the context of a large population sample.

In terms of experimental studies, clinical trials in obese individuals with type 2 diabetes found that leptin therapy was ineffective in improving type 2 diabetes and insulin resistance. These results contradicted pre-clinical trial studies in mouse models that showed improvement in insulin resistance with leptin [28, 40–43]. A limited number of observational population studies assessed the relationship of leptin with incident type 2 diabetes. In a study in Mauritius, high leptin levels were associated with the future development of type 2 diabetes. The association was independent of other factors in men but not in women [5]. Similarly, among Japanese Americans, increased baseline leptin levels were associated with increased risk of developing type 2 diabetes in men but not in women [44]. The study also found that adjustment for insulin resistance abolished the association between leptin and type 2 diabetes, an indication that the association between leptin and incident type 2 diabetes was mediated by insulin resistance [45]. These results are consistent with the results of our findings. Nevertheless, differences by sex in body fat composition—with women generally having a higher body fat percentage and, thus, higher leptin levels—may explain why the association between leptin and type 2 diabetes appears in men but not in women. While it is well-known that circulating leptin levels in the blood are related to body fat composition, our study confirms that the distribution of obesity, rather than overall obesity, is critical for the development of insulin resistance and incident type 2 diabetes mellitus. Further, since the association between leptin and incident type 2 diabetes was found in men but not in women and this association was mediated by insulin resistance, the relationship is likely a function of visceral obesity that resembles the body shape of men rather than women.

In the Sandy Lake Health and Diabetes Project cohort, the association between high leptin at baseline with an increased risk of incident type 2 diabetes disappeared after adjustment for either waist circumference or BMI [46]. Furthermore in the Hoorn study, insulin resistance was a main determinant of leptin levels [47]. Similarly in a Western Samoan population, leptin levels were found to be correlated with fasting insulin concentration and insulin concentration two hours after a glucose load [48]. Nevertheless, in a study from India, lower serum leptin levels in those with type 2 diabetes were partially due to increased waist circumference, decreased BMI, and male sex [49]. In contrast to these studies, our investigation showed that BMI did not change the association between leptin and type 2 diabetes, but that abdominal obesity did. Thus, our study provides useful information regarding the expected differential associations between leptin and type 2 diabetes among those with abdominal obesity. The association between leptin and type 2 diabetes is not only mediated through insulin resistance, but it is present exclusively among those without abdominal obesity. Thus, it can be concluded that the presence of abdominal obesity negates the association between leptin and type 2 diabetes. The opposite is true for adiponectin – type 2 diabetes relationship, as the inverse association was present only among abdominally obese participants. Our results should be interpreted with caution, as we conducted several stratified analyses, the power is reduced despite our initial large sample size. As well some findings may be spurious due to multiple testing.

Due to the study design and population, our findings cannot be generalized to non-African American individuals. As in any observational study, it is limited by residual confounding, although we accounted for the majority of putative confounders. A major strength of our investigation is that the Jackson Heart Study is the largest ongoing cohort of African Americans with high-quality measurements, data collection, and event surveillance.

## Conclusions

In conclusion, in our African American sample, adiponectin was inversely associated with incident type 2 diabetes, while leptin’s direct association with diabetes was mediated by insulin resistance and present only among men and abdominally non-obese participants. The inverse association of adiponectin with type 2 diabetes was present only among abdominally obese participants and persisted after adjustment for HOMA-IR. No interaction between leptin and adiponectin was present. Our results should inform future clinical trials that aim to reduce the burden of type 2 diabetes through the modification of serum levels of leptin and adiponectin and add (although non-experimentally) to the debate regarding the differential effects of leptin on type 2 diabetes (i.e., the ‘leptin resistance’ phenomenon present among diabetic obese individuals).

## Data Availability

Data are available from the JHS Steering Committee after applicable approvals.

## Abbreviations

BMI: body mass index
CI: confidence interval
HbA1c: hemoglobin A1c
HDL: high-density lipoprotein cholesterol
HOMA-IR: homeostasis model assessment – insulin resistance
HR: hazard ration
LDL: low-density lipoprotein cholesterol
n: number of participants
q1 to q4: quartiles 1 through 4
T2D: type 2 diabetes
TC: total cholesterol

## References

[1] Smyth S, Heron A (2006). Diabetes and obesity: the twin epidemics. Nat Med.

[2] Global Burden of Disease Study C (2015). Global, regional, and national incidence, prevalence, and years lived with disability for 301 acute and chronic diseases and injuries in 188 countries, 1990-2013: a systematic analysis for the global burden of disease study 2013. Lancet..

[3] National Diabetes Statistics Report. In: Centers for Disease Control and Prevention USDoHaHS, editor. Centers for Disease Control and Prevention. Atlanta; 2017. p. 20. https://www.cdc.gov/diabetes/data/statistics/statistics-report.html. Accessed 28 Feb 2020.

[4] Geiss LS, Wang J, Cheng YLJ (2014). (2014) prevalence and incidence trends for diagnosed diabetes among adults aged 20 to 79 years, United States, 1980-2012. J Am Med Assoc.

[5] Soderberg S, Zimmet P, Tuomilehto J (2007). Leptin predicts the development of diabetes in Mauritian men, but not women: a population-based study. Int J Obes.

[6] Schmidt MI, Duncan BB, Vigo A (2006). Leptin and incident type 2 diabetes: risk or protection?. Diabetologia.

[7] Gao Y, Yang Q, Zhou J, Chen L (2000). Association between leptin, insulin, and body fat distribution in 2-diabetes mellitus. Ann N Y Acad Sci.

[8] Denroche HC, Huynh FK, Kieffer TJ (2012). The role of leptin in glucose homeostasis. J Diabetes Investig.

[9] D'Souza AM, Neumann UH, Glavas MM, Kieffer TJ (2017). The glucoregulatory actions of leptin. Mol Metab.

[10] Fernandez-Formoso G, Perez-Sieira S, Gonzalez-Touceda D, Dieguez C, Tovar S (2015). Leptin, 20 years of searching for glucose homeostasis. Life Sci.

[11] Perry RJ, Petersen KF, Shulman GI (2016). Pleotropic effects of leptin to reverse insulin resistance and diabetic ketoacidosis. Diabetologia.

[12] Toyoshima Y, Gavrilova O, Yakar S (2005). Leptin improves insulin resistance and hyperglycemia in a mouse model of type 2 diabetes. Endocrinology.

[13] Singh P, Sharma P, Sahakyan KR (2016). Differential effects of leptin on adiponectin expression with weight gain versus obesity. Int J Obes.

[14] Carpenter MA, Crow R, Steffes M (2004). Laboratory, reading center, and coordinating center data management methods in the Jackson heart study. Am J Med Sci.

[15] Taylor HA (2005). The Jackson Heart Study: an overview. Ethn Dis.

[16] Liu J, Butler KR, Buxbaum SG, Sung JH, Campbell BW, Taylor HA (2010). Leptinemia and its association with stroke and coronary heart disease in the Jackson heart study. Clin Endocrinol.

[17] Bidulescu A, Liu J, Musani SK (2011). Association of adiponectin with left ventricular mass in blacks: the Jackson heart study. Circ Heart Fail.

[18] Shand B, Elder P, Scott R, Frampton C, Willis J (2006). Biovariability of plasma adiponectin. Clin Chem Lab Med.

[19] Davis SK, Gebreab SY, Xu R (2015). Association of adiponectin with type 2 diabetes and hypertension in African American men and women: the Jackson heart study. BMC Cardiovasc Disord.

[20] Matthews DR, Hosker JP, Rudenski AS, Naylor BA, Treacher DF, Turner RC (1985). Homeostasis model assessment: insulin resistance and beta-cell function from fasting plasma glucose and insulin concentrations in man. Diabetologia.

[21] Valeri L, Vanderweele TJ (2013). Mediation analysis allowing for exposure-mediator interactions and causal interpretation: theoretical assumptions and implementation with SAS and SPSS macros. Psychol Methods.

[22] Valeri L, VanderWeele TJ (2015). SAS macro for causal mediation analysis with survival data. Epidemiology.

[23] Shpakov AO (2014). The role of alterations in the brain signaling systems regulated by insulin, IGF-1 and leptin in the transition of impaired glucose tolerance to overt type 2 diabetes mellitus. Tsitologiia.

[24] Meek TH, Morton GJ (2012). Leptin, diabetes, and the brain. Indian J Endocrinol Metab.

[25] Barber M, Kasturi BS, Austin ME, Patel KP, MohanKumar SM, MohanKumar PS (2003). Diabetes-induced neuroendocrine changes in rats: role of brain monoamines, insulin and leptin. Brain Res.

[26] Turer AT, Scherer PE (2012). Adiponectin: mechanistic insights and clinical implications. Diabetologia.

[27] Yamauchi T, Kamon J, Waki H (2003). Globular adiponectin protected Ob/Ob mice from diabetes and ApoE-deficient mice from atherosclerosis. J Biol Chem.

[28] Coppari R, Bjorbaek C (2012). Leptin revisited: its mechanism of action and potential for treating diabetes. Nat Rev Drug Discov.

[29] Myers MG, Cowley MA, Munzberg H (2008). Mechanisms of leptin action and leptin resistance. Annu Rev Physiol.

[30] Al-Hamodi Z, Al-Habori M, Al-Meeri A, Saif-Ali R (2014). Association of adipokines, leptin/adiponectin ratio and C-reactive protein with obesity and type 2 diabetes mellitus. Diabetol Metab Syndr.

[31] Scotece M, Conde J, Lopez V (2014). Adiponectin and leptin: new targets in inflammation. Basic Clin Pharmacol Toxicol.

[32] Duncan BB, Schmidt MI, Pankow JS (2004). Adiponectin and the development of type 2 diabetes: the atherosclerosis risk in communities study. Diabetes.

[33] Bi X, Loo YT, Henry CJ (2018). Does circulating leptin play a role in energy expenditure?. Nutrition.

[34] Garaulet M, Perex-Llamas F, Fuente T, Zamora S, Tebar FJ (2000). Anthropometric, computed tomography and fat cell data in an obese population: relationship with insulin, leptin, tumor necrosis factor-alpha, sex hormone-binding globulin and sex hormones. Eur J Endocrinol.

[35] Lonnqvist F, Wennlund A, Arner P (1997). Relationship between circulating leptin and peripheral fat distribution in obese subjects. Int J Obes Relat Metab Disord.

[36] Minocci A, Savia G, Lucantoni R (2000). Leptin plasma concentrations are dependent on body fat distribution in obese patients. Int J Obes Relat Metab Disord.

[37] Staiger H, Tschritter O, Machann J (2003). Relationship of serum adiponectin and leptin concentrations with body fat distribution in humans. Obes Res.

[38] Ostlund RE, Yang JW, Klein S, Gingerich R (1996). Relation between plasma leptin concentration and body fat, gender, diet, age, and metabolic covariates. J Clin Endocrinol Metab.

[39] Genske F, Kuhn JP, Pietzner M (2018). Abdominal fat deposits determined by magnetic resonance imaging in relation to leptin and vaspin levels as well as insulin resistance in the general adult population. Int J Obes.

[40] Berglund ED, Vianna CR, Donato J (2012). Direct leptin action on POMC neurons regulates glucose homeostasis and hepatic insulin sensitivity in mice. J Clin Invest.

[41] Coppari R, Ichinose M, Lee CE (2005). The hypothalamic arcuate nucleus: a key site for mediating leptin's effects on glucose homeostasis and locomotor activity. Cell Metab.

[42] Cummings BP, Bettaieb A, Graham JL (2011). Subcutaneous administration of leptin normalizes fasting plasma glucose in obese type 2 diabetic UCD-T2DM rats. Proc Natl Acad Sci U S A.

[43] Moon HS, Matarese G, Brennan AM (2011). Efficacy of metreleptin in obese patients with type 2 diabetes: cellular and molecular pathways underlying leptin tolerance. Diabetes.

[44] McNeely MJ, Boyko EJ, Weigle DS (1999). Association between baseline plasma leptin levels and subsequent development of diabetes in Japanese Americans. Diabetes Care.

[45] Wannamethee SG, Lowe GD, Rumley A, Cherry L, Whincup PH, Sattar N (2007). Adipokines and risk of type 2 diabetes in older men. Diabetes Care.

[46] Ley SH, Harris SB, Connelly PW (2008). Adipokines and incident type 2 diabetes in an aboriginal Canadian [corrected] population: the Sandy Lake health and diabetes project. Diabetes Care.

[47] Ruige JB, Dekker JM, Blum WF (1999). Leptin and variables of body adiposity, energy balance, and insulin resistance in a population-based study. The Hoorn Study Diabetes Care.

[48] Zimmet P, Hodge A, Nicolson M, et al. (1996) serum leptin concentration, obesity, and insulin resistance in Western Samoans: cross sectional study. BMJ 1996;313:965–969. 10.1136/bmj.313.7063.965 PMC2352310 8892415

[49] Marita AR, Sarkar JA, Rane S (2005) type 2 diabetes in non-obese Indian subjects is associated with reduced leptin levels: study from Mumbai, Western India. Mol Cell Biochem 2005;275:143–151. 10.1007/s11010-005-1204-7 16335794

